# Access and outcomes of general practitioner obstetrician (rural generalist)‐supported birthing units in Queensland

**DOI:** 10.1111/ajr.12593

**Published:** 2020-01-05

**Authors:** Debra Tennett, Lauren Kearney, Mary Kynn

**Affiliations:** ^1^ Gympie Hospital Sunshine Coast Hospital and Health Service Gympie Queensland Australia; ^2^ School of Nursing, Midwifery and Paramedicine University of the Sunshine Coast Maroochydore DC Queensland Australia; ^3^ School of Health and Sports Sciences University of the Sunshine Coast Maroochydore DC Queensland Australia

**Keywords:** access, maternity, obstetrics, risk, rural

## Abstract

**Objective:**

To describe characteristics and outcomes of women birthing within GP‐obstetrician (rural generalist) supported rural (level 3) obstetric units in Queensland.

**Design:**

Retrospective descriptive study.

**Setting:**

21 GP‐obstetrician supported birthing units in Queensland.

**Participants:**

Women (n = 3111) birthing from January 2017 to December 2017.

**Main outcome measures:**

Patient, pregnancy and labour characteristics and key maternal and neonatal outcomes routinely recorded in the Queensland Perinatal Data Collection and Queensland Hospital Admitted Patient Data Collection were compared to Queensland public hospital aggregate data.

**Results:**

Women birthing in rural maternity units were significantly more likely to be Aboriginal or Torrs Strait Islander (16% v 9%), < 20 years old (7% v 4%), term deliveries (96% v 91%), achieve spontaneous onset of labour (67% v 51%), and birth (71% v 60%) (*p*<0.001) compared with all Queensland public hospitals. They were significantly less likely to be nulliparous (36% v 40%), use pharmacological analgesia (65% v 69%), or have continuous electronic fetal monitoring in labour (54% v 66%) (*p*<0.001). Neonatal outcomes were comparable; with no significant difference in stillbirth rate between rural units and all Queensland public hospitals (4.8 v 7.3 per 1000 births). Precipitate delivery was the most common labour complication (36% v 33%) (*p*<0.001).

**Conclusion:**

GP‐obstetrician (rural generalist) supported rural birthing units in Queensland provide important access for low and medium risk women to deliver locally, with strong indicators of quality and safety.

**What is already known on this subject:**

- A 20‐year old study suggested that lower volume of births is not associated with adverse outcomes for low risk women birthing in Australia.
- There has been no recent published information on characteristics or outcomes associated with different volumes of level 3 GP‐obstetrician (rural generalist‐supported) maternity units, which provide intrapartum services for a wide catchment and mixed risk population.
- Such information is timely with the National Rural Generalist Pathway currently under development.

**What this study adds:**

- This paper identifies patient characteristics and key birthing outcomes of GP‐obstetrician (rural generalist‐supported) level 3 maternity units by service volume (<100, 100‐199, ≥200 deliveries per year).
- Compared with average figures from all Queensland public hospitals, level 3 maternity units demonstrate reassuring outcomes despite mixed risk cohorts.

## 1 INTRODUCTION

Thirty per cent of Queensland's population is dispersed throughout rural and remote areas and deserve safe and high‐quality birthing services as close to home as possible.^1^ The tendency towards centralising health services in many developed nations, including Australia, has resulted in rural and remote maternity unit closures. In Queensland, from 1992 to 2011 there was a net reduction of 36 maternity units (28%), effectively increasing the distance to birthing units for rural families.^2^ Closures were linked to issues of rurality, medical and midwifery workforce shortages, safety and quality concerns, perceived higher costs^2^ and disconnected localised planning and reactionary decision‐making.^3^ This trend has been met with local community protest and concern.^4^

The loss of rural maternity units can have substantial consequences for communities already known to suffer poor social determinants of health.^5^ During the 20‐year maternity unit closure period in Queensland, the rate of babies born before arrival (BBA) doubled, reaching 429 births by 2011, and was highest in inner and outer regional areas.^2^ These findings are consistent with international research, and a large population study in British Columbia between 2000 and 2004 found that the odds of having an unplanned out of hospital birth is 6.41 (95% CI 3.69, 11.28) for women 1‐2 hours away from services with statistically significant increases in perinatal mortality for newborns whose mothers reside more than 4 hours from services, as well as induction rates for logistical reasons highest for women located 2‐4 hours from services.^6^

Distance from a maternity unit not only impacts on delivery plans but also increases distress for those having to travel large distances^7^ and for those labouring en route.^4^ Aboriginal and Torres Strait Islander women, 55% of whom live in outer regional and remote areas of Australia,^8^ report high rates of pregnancy stress and low levels of birthing choice.^9^ Many rural and remote mothers travel to birth “off country” at between 36 and 38 weeks’ gestation,^10^ disrupting their other children, partner's work and increasing emotional and financial strain related to isolation, travel and accommodation.^9^

The Queensland government implemented the Queensland Health Rural Generalist Pathway (QRGP) in 2007 as a long‐term workforce initiative to reverse the downward trend in health service provision, including maternity services, to Queensland's rural and remote communities.^11^ This QRGP initiative was specifically designed to train and support rural generalist doctors (qualified and recognised GPs with advanced skills including obstetrics, anaesthetics and emergency medicine) to meet the needs of Queensland's rural and remote communities.^12^ The National Maternity Services Plan (2010) further recognised the importance of maternity services within the Australian health system and identified four priority areas (access, service delivery, workforce and infrastructure) requiring urgent attention.^13^ An expanded role for midwives as well as procedural GP‐obstetricians and anaesthetists was identified as a key factor for improving services in rural Australia.^14^

Since the commencement of the QRGP, there has been a noticeable increase in numbers of rural generalist doctor training and staying in Queensland rural practice with corresponding reductions in rural vacancies^15^ and reopening of four rural maternity units. A formal external review by Ernst and Young in 2013 found the QRGP to be an effective and sustainable training pathway providing a solution to the rural medical workforce issues in Queensland, with a conservative estimate on return on investment ratio in the vicinity of 1.2.^16^

Indeed, available literature suggests that historical concerns about rural maternity unit safety can be unsubstantiated.^17^ A large Australian population‐based study from 1999 to 2001 found lower service volumes were not associated with adverse outcomes for low‐risk women (defined as women aged 20‐34, without pre‐existing medical conditions and no obstetric complications, such as gestational diabetes or hypertension) compared with hospitals with >2000 births per year.^17^ However, this study is relatively dated, and the low volume births included primary maternity units (PMU) which do not have onsite caesarean capability. In addition, a retrospective Queensland study published in 2015 explored three years of activities and outcomes for a PMU located 1 hour from a unit with surgical capability and found clinical outcomes were comparable or better than Queensland wide data.^18^ However, despite being recipients of substantial government training and support, and with the national Rural Generalist Training Pathway on the national agenda in Australia, characteristics and outcomes of qualified GP‐obstetrician (rural generalist‐supported) birthing units remain to be explored.

This study therefore aimed to examine maternal characteristics and key outcomes for GP‐obstetrician (rural generalist) supported rural maternity units in 2017 compared with all Queensland public hospitals, as well as examine the same rural units by volume of delivery (<100 births, 100‐199 births, ≥200 births). The ensuing patient characteristics might offer some insights into risk profiling of women delivering in rural maternity units compared with the Queensland average. The selected outcomes, compared with the Queensland average, may reflect the proficiency of operational frameworks underpinning GP‐obstetrician (rural generalist‐supported) maternity units that might give opportunity to reflect on the Queensland Rural Generalist Pathway and the future national direction.

## 2 METHODS

This study employed a retrospective design. In 2017, Queensland had 39 public hospital maternity units with 22 facilitating less than 500 births per year.^19^ These level 3 maternity services are predominantly supported by GP‐obstetricians (rural generalists) rather than obstetricians and do not have a special care nursery or specialist paediatric cover.^20^ Only centres with a stable GP‐obstetrician workforce in 2017 were included, determined by a phone call to each maternity unit. One maternity unit was excluded due to a heavy reliance of locums in 2017. Data were collected from the remaining 21 GP‐obstetrician (rural generalist) supported maternity units, referred to as rural units for the remainder of this article, via the Queensland Perinatal Data Collection and Queensland Hospital Admitted Patient Data Collection. The included units were not necessarily operational for the entire year. All women who gave birth between January 2017 and December 2017 were included.

Data coalesced into three categories: mother's details (place of birth, antenatal transfer, including reason for transfer and time of transfer, Indigenous status, age, pregnancy history, body mass index, antenatal care history, current medical conditions and complications arising during pregnancy); labour and birth (location, onset of labour, labour length, mode of birth and reasons for, perineal injury, pharmacological analgesia, electronic foetal monitoring and intrapartum complications); and baby (Indigenous status, birth‐weight, gestation, plurality, APGARS [newborn wellbeing measure], neonatal resuscitation, oral fluids prior to discharge).

Summary measures were calculated for each of the above data categories and compared with Queensland public hospital averages (whole of state) provided by Statistical Services Branch, Queensland Health, using a chi‐squared test. Rural units were divided into different volumes of delivery, <100, 100‐199 and >200 deliveries per year and compared using a chi‐squared test. The level for significance was set at *α* = 0.001 to account for multiple testing. A multivariate analysis was not conducted as this study was descriptive in design and individual risk profiles for the comparison group were unknown.

### 2.1 Ethics approval

The study had ethical approval from The Prince Charles Hospital Human Research Ethics Committee [HREC/18/QPCH/218].

## 3 RESULTS

**Figure 1:**
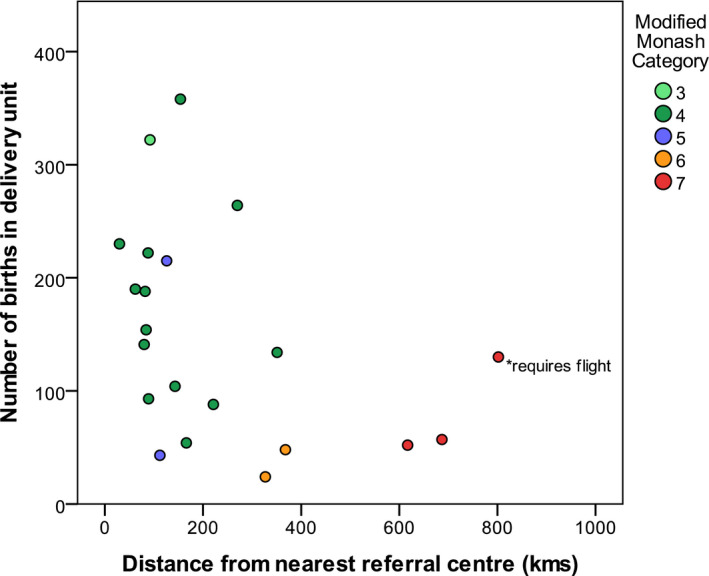
Distance from referral centre (minutes by road) by size of maternity unit

From January 2017 to December 2017, there were 3116 babies born to 3111 mothers in the 21 rural units. Rural maternity units’ distance from corresponding secondary or tertiary‐level referral centre varied from 50 to 686 km by road, with one centre including a flight (Figure 1). Modified Monash categories (2019) derived using 2016 ABS data for the rural hospitals ranged from 3 to 7 (Figure 1).

### 3.1 Maternal characteristics

Although the majority of mothers delivering in rural units had low‐risk characteristics, some characteristics known to be associated with increased pregnancy risk were higher than for Queensland public hospital averages. These characteristics included birthing for more Aboriginal and Torres Strait Islander women, mothers who were overweight and women less than 20 years of age, when compared with Queensland public hospital averages (Table 1).

**Table 1:** Characteristics of mothers birthing in Queensland rural GP‐obstetrician supported rural units in 2017, by volume of deliveries, compared with state‐wide data for the same period. Percentages are calculated out of all mothers

Characteristics	Rural units				All QLD public hospitals
	<100 births n = 459	100‐199 births n = 1041	>200 births n = 1611	Total n = 3111	Total N = 44 329
	n (%)	n (%)	n (%)	n (%)	%
Indigenous	80 (17)	226 (22)	177 (11)	483 (16)	9
BMI^a^					
Underweight	11 (2)	59 (6)	86 (5)	156 (5)	6
Normal	228 (50)	458 (45)	786 (49)	1472 (47)	47
Overweight	132 (29)	271 (27)	419 (26)	822 (26)	23
Obese all	86 (19)	234 (23)	304 (19)	624 (20)	24
Obese class I	58 (13)	154 (15)	199 (12)	411 (13)	12
Obese class II	26 (6)	64 (6)	8 (5)	175 (6)	6
Obese class III	2 (1)	13 (1)	17 (1)	32 (1)	4
Not stated	2 (1)	19 (2)	16 (1)	37 (1)	2
Antenatal visits					
0‐4	16 (3)	55 (5)	85 (5)	156 (5)	6
5+	443 (97)	984 (95)	1525 (95)	2952 (95)	94
Age group^a^					
<20	33 (7)	85 (8)	100 (6)	218 (7)	4
20‐34	389 (85)	834 (80)	1302 (81)	2525 (81)	79
35+	37 (8)	122 (12)	208 (13)	367 (12)	17
Previous pregnancies					
0	130 (28)	281 (27)	423 (26)	834 (27)	29
1‐3	267 (58)	585 (56)	976 (61)	1828 (59)	57
4+	62 (14)	175 (17)	212 (13)	449 (14)	14
Parity^a^					
0	160 (35)	367 (35)	595 (37)	1122 (36)	40
1‐3	274 (60)	589 (56)	930 (58)	1793 (58)	55
4+	25 (5)	85 (8)	86 (5)	196 (6)	6

a Significant difference (*P* < .001) between total rural proportions and QLD wide proportions, chi‐squared test.

### 3.2 Key outcomes

#### 3.2.1 Labour and birth outcomes

Rural birthing mothers were significantly more likely to birth at term, have spontaneous onset of labour and achieve a vaginal birth, than Queensland averages. Rural birthing mothers were significantly less likely to have cardiotocography (CTG) in labour or use pharmacological analgesia in labour (Table 2).

**Table 2:** Labour and birth outcomes in Queensland GP‐obstetrician supported rural units by volume of deliveries compared with state‐wide births for the same period. Percentages are calculated out of all births unless otherwise stated in parentheses

Characteristics	Rural units				All QLD public hospitals
	<100 births n = 459	100‐199 births n = 1043	≥200 births n = 1614	Total n = 3116	Total N = 44 966
	n (%)	n (%)	n (%)	n (%)	%
Gestation in weeks^a^					
37+	447 (97)	1003 (96)	1548 (96)	2998 (96)	91
<37 wk	12 (3)	40 (4)	66 (4)	118 (4)	9
Live births					
Live births	456 (99)	1037 (100)	1608 (100)	3101 (100)	99
Stillbirths	3 (1)	6 (1)	6 (<1)	15 (<1)	1
Labour onset^a^					
Spontaneous	312 (68)	725 (70)	1041 (64)	2078 (67)	51
Induced	96 (21)	196 (19)	378 (23)	670 (22)	31
No labour (caesarean)	51 (11)	122 (12)	195 (12)	368 (12)	17
Delivery method^a^					
Forceps	2 (0)	6 (1)	7 (0)	15 (<1)	3
Vacuum	23 (5)	33 (3)	104 (6)	160 (5)	7
Lower Segment Caesarean Section (LSCS)	106 (23)	256 (25)	366 (23)	728 (23)	30
Classical caesarean section	0 (0)	1 (0)	0 (0)	0	<1
Vaginal (non‐instrumental)	328 (71)	747 (72)	1137 (70)	2212 (71)	60
Cardiotocography in labour (mothers) a					
No	206 (45)	522 (50)	697 (43)	1425 (46)	34
Yes	253 (55)	519 (50)	914 (57)	1686 (54)	66
Use of analgesia during labour^a^					
No	142 (31)	428 (41)	523 (32)	1093 (35)	31
Yes	317 (69)	615 (59)	1091 (68)	2023 (65)	69
Epidural/caudal/Spinal (CSE)	106 (23)	220 (21)	384 (24)	710 (23)	27
Baby weight^a^					
<2.5 kg	12 (3)	36 (3)	43 (3)	91 (3)	8
2.5‐4 kg	388 (85)	894 (86)	1372 (85)	2654 (85)	82
>4 kg	59 (13)	113 (11)	199 (12)	371 (12)	10
Apgar at 5 min (live births)					
<7	8 (2)	24 (2)	24 (2)	56 (2)	2
7‐10	448 (98)	1013 (97)	1584 (98)	3045 (98)	98
Resuscitation (live births)^a^					
No	410 (90)	914 (88)	1423 (89)	2747 (89)	78
Yes	46 (10)	123 (12)	185 (12)	354 (11)	22
Bag and mask b	16 (4)	59 (6)	85 (5)	160 (5)	9
Intermittent Positive Pressure Ventilation (IPPV) via Endotracheal Tube (ETT) b	2 (<1)	12 (1)	14 (1)	28 (1)	1
Facial oxygen b	19 (4)	35 (3)	75 (5)	129 (4)	6
External cardiac massage^b^	1 (<1)	8 (1)	3 (<1)	12 (<1)	<1
Other^b^, ^c^	20 (4)	75 (7)	119 (7)	214 (7)	17
Transfer (mothers)^a^					
Not transferred	455 (99)	1013 (97)	1558 (97)	3026 (97)	94
Prior to onset of labour	3 (1)	14 (1)	21 (1)	38 (1)	5
During labour	1 (<1)	14 (1)	32 (2)	47 (2)	1

a Significant difference (*P* < .001) between total rural proportions and QLD wide proportions, chi‐squared test.

b Resuscitation allowed reporting of multiple items and cannot be cumulated

c Other includes suction (eg, oral and pharyngeal), suction of meconium (eg, oral and pharyngeal), suction of meconium via ETT, narcotic antagonist injection, adrenalin/sodium bicarbonate, other drugs, other stimulations

Neonatal outcomes were comparable between groups, with no evidence of harm afforded to babies born in smaller units (Table 2).

#### 3.2.2 Selected pregnancy, labour and birth complications

There were more precipitate births associated with the rural units compared with Queensland overall and increased uterine inertia; however, for all other labour and birth complications, the proportions were either similar or less than that of Queensland overall (Table 3). The rate of second birth being vaginal after first birth caesarean (VBAC) was similar for rural units compared with Queensland overall with 9 out of 21 units having VBACs recorded; 7 out of the 9 hospitals providing VBACs were located within 2 hours of their referral hospital, and 1 was located >4 hours from a referral hospital.

**Table 3:** Selected pregnancy, labour and birth complications comparing different sized Queensland rural GP‐obstetrician supported units with state‐wide figures for the same period. Percentages are calculated out of all births. More than one complication might have been recorded for each birth, so the numbers cannot be cumulated

	Rural units				All QLD public hospitals
	<100 births n = 459	100‐199 births n = 1043	≥200 births n = 1614	Total n = 3116	Total N = 44 966
	n (%)	n (%)	n (%)	n (%)	%
Pregnancy complications					
Hypertension^a^	16 (3)	42 (4)	63 (4)	121 (4)	7
Type 2 diabetes mellitus	0 (0)	2 (<1)	5 (<1)	7 (<1)	<1
Gestational diabetes mellitus^a^	36 (8)	107 (10)	193 (12)	336 (11)	14
Labour and birth complications					
Precipitate delivery^a^	155 (34)	388 (37)	580 (36)	1123 (36)	33
Foetal distress and/or meconium liquor^a^	44 (10)	171 (16)	250 (15)	465 (15)	22
Primary post‐partum haemorrhage	36 (8)	113 (11)	174 (11)	323 (10)	10
Obstructed labour	23 (5)	73 (7)	102 (6)	198 (6)	6
Previous uterine scar	26 (6)	77 (7)	99 (6)	202 (7)	6
Uterine inertia (incl. failure to progress)^a^	37 (8)	78 (7)	93 (6)	208 (7)	5
Prolonged second stage^a^	19 (4)	32 (3)	49 (3)	100 (3)	4
Cord entanglement with/without compression	3 (1)	15 (1)	15 (1)	33 (1)	3
Breech presentation	6 (1)	13 (1)	13 (1)	32 (1)	2
Retained placenta with/without haemorrhage	4 (1)	20 (2)	19 (1)	43 (1)	1
Maternal distress^a^	0 (0)	9 (1)	5(<1)	14 (<1)	1

a Significant difference (*P* < .001) between total rural proportions and QLD wide proportions, chi‐squared test.

The different sized rural units differed only in the units with 100‐199 births per year having more Aboriginal and/or Torres Strait Islander women, less CTG and less analgesia in labour.

## 4 DISCUSSION

This is the first state‐wide study exploring the characteristics and outcomes for GP‐obstetric (rural generalist) supported birthing units, by service volume, in Queensland since the inception of the Queensland Rural Generalist Pathway over a decade ago: the results are reassuring.

Firstly, it can be appreciated that mothers delivering at GP‐obstetric (rural generalist) supported maternity units are not all low risk, but have a mixed risk profile. Some characteristics associated with poorer outcomes are more prevalent in rural units compared with Queensland average. For example, rural mothers were more likely to be younger than 20, an age that conveys increased risk of adverse maternal perinatal outcomes, including eclampsia, cephalopelvic disproportion, preterm birth, poor foetal growth and low birth‐weight.^21^ Rural units were found to birth a higher proportion Aboriginal and Torres Strait Islanders, who are known to suffer health inequality.^5^ There were more overweight mothers and similar numbers of obese mothers with BMI < 40 compared with Queensland average, characteristics associated with increased pregnancy risks including preeclampsia, gestational diabetes and intrauterine death.^22^ Most rural units have risk stratification policies requiring transfer of patients with BMI > 40 to referral centres for delivery. Australians living in rural and remote areas tend to suffer poor health outcomes compared with those in metropolitan areas, due to social determinants of health.^5^ It is unrealistic to expect all women delivering in rural areas to be low risk. Indeed, rural mothers were more likely to have prior pregnancies reach viable gestational age, perhaps due to differences in choice and/or access to termination, reflecting endemic health inequalities.^23^

Secondly, the results reveal that labour and birth outcomes are reassuring for women birthing in rural units, despite mixed risk profiles. These findings likely reflect suitable triaging and referral processes for higher risk pregnancies where specialised obstetric and/or paediatric support is anticipated, and a tendency to avoid intervention for lower risk pregnancies. Delivery is planned to occur in the most appropriate facility to meet the woman's needs, with antenatal care occurring locally or with the referral centre, sometimes via teleconferencing. Yet the reality is that some “high‐risk” women, for example with severe preeclampsia or BMI > 40, do birth locally, due to either the time critical nature of an emergency presentation or difficulties with transfer. The rural staff must be prepared to handle such emergencies with limited resources, such as access to cross‐matched bloods and theatres not staffed 24 hours a day. The baby is subsequently retrieved as necessary. The outcomes suggest that rural units are managing broad risk birthing cohorts appropriately.

Furthermore, compared with Queensland overall, rural units had similar or fewer labour and birth complications other than an increase in precipitous labour and uterine inertia. Precipitate delivery (labour lasting less than three hours) was the most common labour complication for rural maternity units, perhaps partly explaining the increased rate of BBAs with maternity unit closures, and highlighting the importance of maintaining good access to intrapartum services. Positively, the current Queensland Government has stated that future maternity unit closures will require ministerial approval.^24^

As availability of VBACs is a known source of contention in rural areas, the frequency of these was reviewed. The rate of VBAC was similar between the rural cohort and Queensland average; however, 12 out of 21 units did not have any, and the overall numbers were low. There were 200 women having their second birth whose first birth was by caesarean section, 23 of whom achieved a vaginal birth. The ability of a rural maternity service to offer an intended vaginal birth after caesarean section provides choice for women who want to birth locally that is not limited to repeat caesarean section. Despite all having level 3 service capability, rural units offer varying levels of complexity of care depending on local governance frameworks, some of which might be based on historical factors rather than strong evidence base. While it is unknown whether the VBACs that occurred rurally were planned for local delivery, the volume of delivery and distance from referral centre appear to have some bearing on provision of VBAC.

Overall, this study looked at outcomes inclusive of all patients delivering in rural GP‐obstetrician supported Queensland maternity units in 2017 revealing no obvious concerns. This might reflect the robust rural generalist training in Queensland^12^ along with the availability of professional development programs and federal grants to support upskilling and skill maintenance including Premium Support Scheme^25^ and Rural Procedural Grants Program.^26^ In addition, Queensland Health benefits from robust safety and quality governance framework, which include morbidity and mortality meetings/clinical review committee meetings, investigations of Severity Assessment Code 1 and 2 incidences and use of validated analysis tools to formally review incidents, managed by the Patient Safety and Quality Improvement Service.^27^ Queensland Health also facilitates team‐based professional development programs including Practical Obstetric Multi‐Professional Training (PROMPT), and neonatal resuscitation and ensures competencies are met in intrapartum foetal surveillance^28^ and provide rigorous evidence‐based Maternity and Neonatal Clinical Guidelines.

Even more reassuring in the current credentialing landscape, where procedural numbers are of interest, the different sized rural units appeared remarkably homogenous for most of the characteristics and selected outcomes. These findings are reassuring to child‐bearing women and those working in rural maternity units with <100 births per year and provide evidence to support the current credentialing process for rural generalists and validation of small rural units.

The units included in this study are very heterogeneous in case mix and models of care but this study has grouped them according to their level of service as a point of commonality. Ultimately, even if a woman's care is initially midwifery led, a well communicated multi‐disciplinary team approach involving medical support is often required to manage pregnancies that deviate from “normal,” along with complications and ensure provision of operative delivery. The reassuring outcomes, despite mixed risk profiles and at times challenging working conditions, also reflect the strong and capable teams that are forged in rural maternity units. The smaller staffing numbers and strong community relationships can lead rural maternity units to naturally provide continuity of care/r models, with the benefits this entails.

In conclusion, this research found GP‐obstetricians (rural generalists) effectively support safe and high‐quality maternity care, with lower rates of intervention and equivalent early neonatal outcomes to state‐wide averages, for rural units with varying volumes of deliveries and a mixed risk cohort. The findings of this research can be used to inform future national and international rural generalist developments.

The data used were limited to that available in existing data sets, and this study was conducted over a limited timeframe in a single state, chosen as it has invested in training rural generalists. A large‐scale prospective study comparing rural vs metropolitan birthing outcomes for mothers with different risk profiles might be warranted as the National Rural Generalist Training Pathway rolls out.

## CONFLICT OF INTEREST

Dr Tennett was the recipient of a $5000 research grant from the Rural Generalist Pathway (Queensland Health) to support the undertaking of this study. The authors have no conflicts of interest to declare.

## AUTHORS CONTRIBUTION

All authors made a substantial contribution to this study and approve the final version. DT was involved in conception, design, write‐up; LK was involved in design, write‐up, ethical and governance applications; MK was involved in data coding, analysis and write‐up.

## References

[1] Queensland Treasury . Population Growth Highlights and Trends, Queensland Regions, 2015 edition. Brisbane City, QLD: Queensland Government Statistician’s Office; 2015.

[2] Kildea S , McGhie AC , Gao Y , Rumbold A , Rolfe M . Babies born before arrival to hospital and maternity unit closures in Queensland and Australia. Women Birth. 2015;28(3):236‐245. 25845486 10.1016/j.wombi.2015.03.003

[3] Longman J , Pilcher JM , Donoghue DA , et al. Identifying maternity services in public hospitals in rural and remote Australia. Aust Health Rev. 2014;38(3):337‐344. 24882523 10.1071/AH13188

[4] Dietsch E , Shackleton P , Davies C , Alston M , McLeod M . 'Mind you, there's no anaesthetist on the road': women's experiences of labouring en route. Rural Remote Health. 2010;10(2):1. 20387979

[5] National Rural Health Alliance . Rural and Remote Health. 2017 https://www.aihw.gov.au/reports/rural-health/rural-remote-health/contents/rural-health. Accessed June 27, 2019.

[6] Grzybowski S , Stoll K , Kornelsen J . Distance matters: a population based study examining access to maternity services for rural women. BMC Health Serv Res. 2011;11:147‐147. 21663676 10.1186/1472-6963-11-147 PMC3126704

[7] Patterson JRMP , Foureur MRMP , Skinner JRMP . Remote rural women's choice of birthplace and transfer experiences in rural Otago and Southland New Zealand. Midwifery. 2017;52:49‐56. 28600971 10.1016/j.midw.2017.05.014

[8] Kildea S , Kruske S , Barclay L , Tracy S . ‘Closing the Gap’: how maternity services can contribute to reducing poor maternal infant health outcomes for Aboriginal and Torres Strait Islander women. Rural Remote Health. 2010;10(3):1383. 20707592

[9] Parker S , McKinnon L , Kruske S . 'Choice, culture and confidence': Key findings from the 2012 having a baby in Queensland Aboriginal and Torres Strait Islander survey. BMC Health Serv Res. 2014;14(1):1‐27. 24884930 10.1186/1472-6963-14-196 PMC4012088

[10] Kildea S . Risky business: contested knowledge over safe birthing services for Aboriginal women. Health Sociol Rev. 2006;4:387.

[11] Darling Downs Hospital and Health Service . The Queensland Rural Generalist Program. 2015 http://ruralgeneralist.qld.gov.au/wp-content/uploads/2017/07/2015-lennox-his-rep.pdf. Accessed March 16, 2018.

[12] Sen Gupta T , Manahan D , Lennox D , Taylor N The Queensland Health Rural Generalist Pathway: providing a medical workforce for the bush. Rural Remote Health *.* 2013;13:2319 https://www.rrh.org.au/journal/article/2319. Accessed March 23, 2018. 24033159

[13] Commonwealth of Australia 2009 . National Maternity Services Plan. 2011 http://www.health.gov.au/internet/main/publishing.nsf/Content/maternityservicesplan. Accessed February 16, 2019.

[14] Commonwealth of Australia 2009 . Report of the Maternity Services Review. 2009 http://www.health.gov.au/internet/main/publishing.nsf/Content/maternityservicesreview-report. Accessed February 15, 2019.

[15] Sen Gupta T , Manahan D , Lennox D , Taylor N , Stewart R , Bond D .Queensland Rural Generalist Pathway: impacts on rural medical workforce. 2013. ruralgeneralist.qld.gov.au/wp‐content/uploads/2017/07/paper_Sen‐Gupta‐Tarun.pdf.

[16] Ernst & Young . Evaluation and Investigative Study of the Queensland Rural Generalist Program. 2013 http://ruralgeneralist.qld.gov.au/wp-content/uploads/2017/07/qrgpeval_rpt_feb13.pdf. Accessed March 16, 2018.

[17] Tracy SK , Sullivan E , Dahlen H , Black D , Wang YA , Tracy MB . General obstetrics: does size matter? A population‐based study of birth in lower volume maternity hospitals for low risk women. BJOG. 2006;113(1):86‐96. 10.1111/j.1471-0528.2005.00794.x 16398776

[18] Kruske S , Schultz T , Eales S , Kildea S . A retrospective, descriptive study of maternal and neonatal transfers, and clinical outcomes of a Primary Maternity Unit in rural Queensland, 2009–2011. Women Birth. 2015;28(1):30‐39. 25458610 10.1016/j.wombi.2014.10.006

[19] Queensland Government . Births by hospital. 2018 https://data.qld.gov.au/dataset/births-by-hospital/resource/1585e886-3037-4874-8353-754f271bc226. Accessed 16 March 2018.

[20] Queensland Health . Clinical services capability framework for public and licensed private health facilities v3.2. Brisbane: Queensland Government Department of Health; 2014.

[21] Cavazos‐Rehg P , Krauss M , Spitznagel E , et al. Maternal age and risk of labor and delivery complications. Matern Child Health J. 2015;19(6):1202‐1211. 25366100 10.1007/s10995-014-1624-7 PMC4418963

[22] Stubert J , Reister F , Hartmann S , Janni W . The risks associated with obesity in pregnancy. Deutsch Aerztebl Int. 2018;115(16):276‐283. 10.3238/arztebl.2018.0276 PMC5954173 29739495

[23] Doran FM , Hornibrook J . Barriers around access to abortion experienced by rural women in New South Wales, Australia. Rural Remote Health. 2016;16(1):3538‐3538. 26987999

[24] Maternity report proposes changes for rural mums [press release]. Media Statements: Queensland Government. 2019 http://statements.qld.gov.au/Statement/2019/6/19/maternity-report-proposes-changes-for-rural-mums. Accessed June 19, 2019.

[25] Australian Government Department of Health . Premium Support Scheme (PSS). 2019 https://www1.health.gov.au/internet/main/publishing.nsf/Content/health-medicalindemnity-faq-pss.htm. Accessed August 18, 2019.

[26] Australian Government Department of Human Services . Rural Procedural Grants Program. 2019 https://www.humanservices.gov.au/organisations/health-professionals/services/medicare/rural-procedural-grants-program. Accessed August 18, 2019.

[27] State of Queensland (Queensland Health) . Best practice guide to clinical incident management. 2014 https://clinicalexcellence.qld.gov.au/sites/default/files/2018-01/clinicalincidentguide.pdf

[28] State of Queensland (Queensland Health) . Guideline supplement: Intrapartum fetal surveillance (IFS). 2018 https://clinicalexcellence.qld.gov.au/sites/default/files/2018-01/clinicalincidentguide.pdf

